# Deep Granuloma Annulare After Scalp Infection: Wolf’s Isotopic Response in a Pediatric Patient

**DOI:** 10.7759/cureus.96166

**Published:** 2025-11-05

**Authors:** Juliana M O'Reilly, Henok Eskinder, Elaine Keung, Luke Bloomquist, Shena Kravitz

**Affiliations:** 1 Graduate Medical Education, Walter Reed National Military Medical Center, Bethesda, USA; 2 Pathology, Walter Reed National Military Medical Center, Bethesda, USA; 3 Dermatopathology, Walter Reed National Military Medical Center, Bethesda, USA; 4 Dermatology, Landstuhl Regional Medical Center, Landstuhl, DEU; 5 Dermatology, Madigan Army Medical Center, Tacoma, USA

**Keywords:** deep granuloma annulare, histology, pediatric, pseudorheumatoid nodule, wolf's isotopic response

## Abstract

We present a case of a pediatric patient with recurrent flares of deep granuloma annulare (GA) largely localized to the right scalp and face, corresponding to the area of a previously resolved scalp infection. This clinical pattern is suggestive of Wolf’s isotopic response, a phenomenon that has been rarely reported in children. To our knowledge, this may represent one of the first documented cases of deep GA manifesting as Wolf’s isotopic response in a pediatric patient.

## Introduction

Wolf’s isotopic response, first described in 1955, represents a reaction where a new skin disorder occurs at the same site of another resolved and unrelated skin condition [1]. This phenomenon is largely reported in adults, most commonly following herpes zoster or herpes simplex infections as the initial skin disorder [1]. The secondary conditions are predominantly granulomatous reactions and malignancies [1]. Granuloma annulare (GA) is the most commonly reported granulomatous reaction seen in Wolf’s isotopic response [1]. GA is a benign non-infectious granulomatous dermatosis with various subtypes. Localized GA is the most common subtype and is usually characterized by annular reddish-brown papules and plaques. Deep GA, however, presents as subcutaneous nodules and is more common in children [2]. Deep GA is often idiopathic, and local recurrence is common [2]. Though GA as a manifestation of this reaction has been well documented in cases of postherpetic reactions in adults, the case we discuss in this report represents one of the first documented instances of Wolf’s isotopic response presenting with recurrent deep GA in a pediatric patient [3,4].

## Case presentation

A 13-year-old immunocompetent male presented to the dermatology clinic with recurrent crops of subcutaneous nodules generally located on the right scalp and face. This distribution was largely localized to the area previously treated for cellulitis on the scalp seven years prior. At the age of six years, the patient presented to his pediatrician with a fever and a 1.5-2 cm erythematous nodule that was tender to palpation on the right temporo-occipital scalp. The patient was clinically diagnosed with cellulitis and treated with a 10-day course of oral cephalexin, with clinical improvement in the fever, and decreased tenderness and size of the original erythematous scalp nodule.

**Figure 1:**
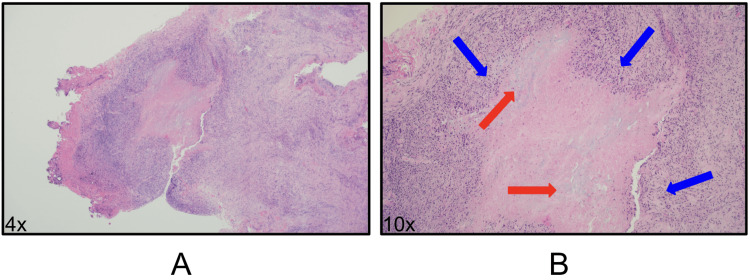
Histology findings - hematoxylin and eosin (H&E) stain (a) At 4x, the hematoxylin and eosin section displays cellular dermal fibroconnective tissue with abundant inflammation and areas of necrobiosis to include foci of collagenolytic debris and mucin. (b) At 10x, the necrobiosis (red arrow) is variably eosinophilic to myxoid and surrounded by palisading lympho-histiocytic inflammatory infiltrate (blue arrow)

**Figure 2:**
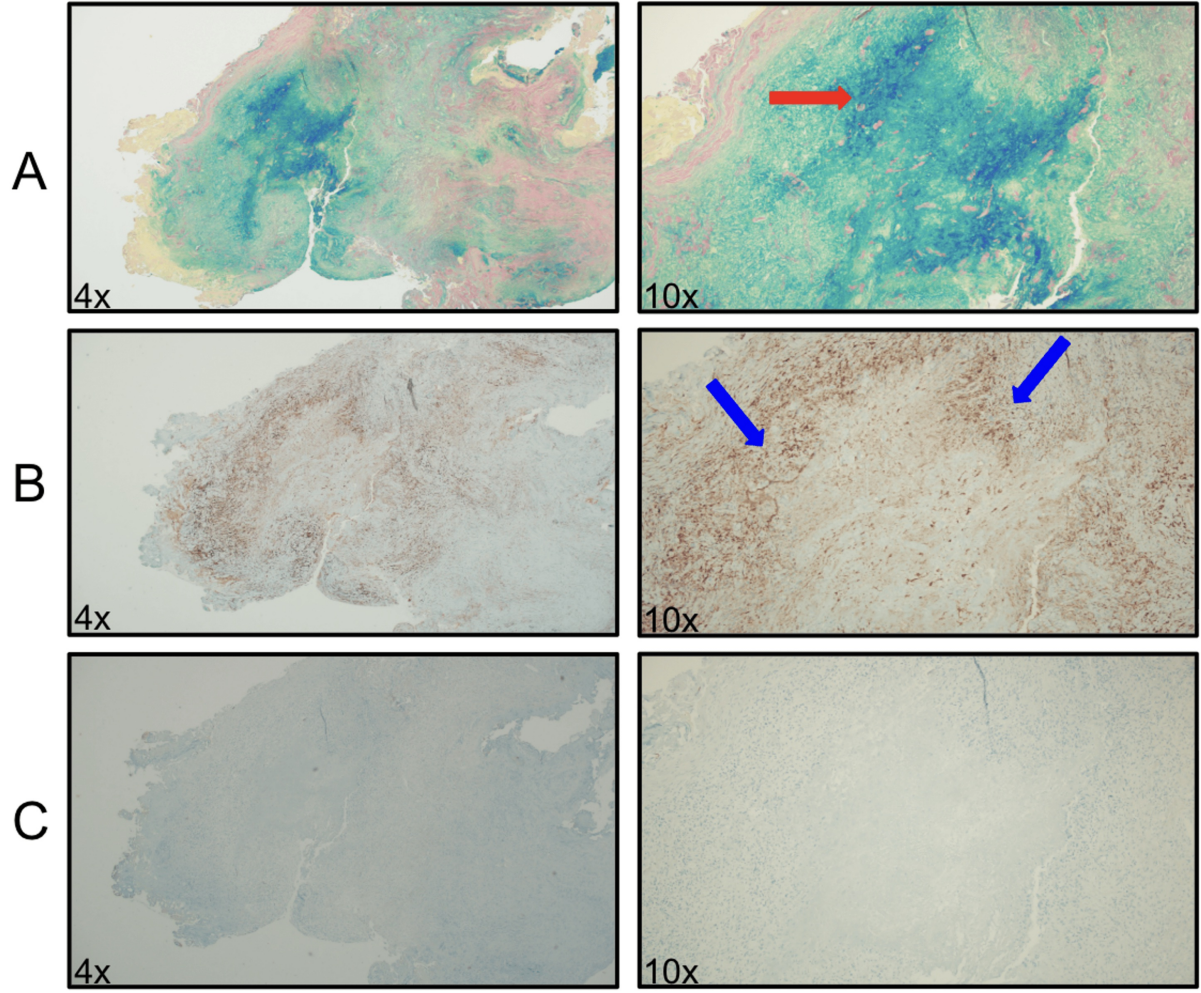
Histology findings - immunohistochemical (IHC) and special stains The following immunohistochemical and special stains are performed on the block: colloidal iron (a), CD163 (b), and AE1/AE3 (c). (a) At 10x, the colloidal iron stain highlights mucin within the foci of necrobiosis (red arrow). (b) At 10x, CD163 stains a predominant population of histiocytes present throughout the specimen and highlights the primary focus of necrobiosis with a rim of positive staining in a palisading pattern surrounding the collagenolytic debris (blue arrow). (c) AE1/AE3 displays a negative staining pattern throughout the specimen and is performed to help rule out a possible differential diagnosis of epithelioid sarcoma

Shortly after, he developed a scaly plaque on the right temporo-occipital scalp that improved with topical clotrimazole 1% cream and selenium sulfide 2.5% lotion. Biopsy and cultures were not collected at the time of initial management. A few months later, the patient developed asymptomatic subcutaneous nodules on the right lateral forehead and left orbital rim. An excisional biopsy of the right lateral forehead nodule revealed a palisading granulomatous dermatitis with centralized deep dermal mucin and necrobiosis without significant fibrosis, plasma cells, or multinucleated giant cells, consistent with the diagnosis of deep GA (Figures 1, 2).

**Figure 3:**
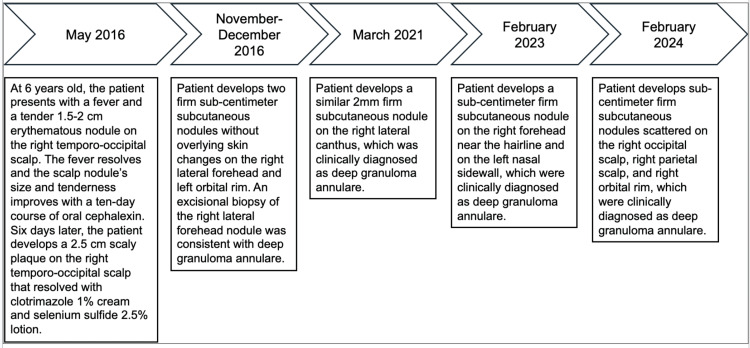
Timeline of clinical events Timeline of events from the initial treatment of the suspected scalp cellulitis, with documented instances of recurrent deep granuloma annulare in the patient’s medical record

Since the initial appearance of the deep GA nodules, the patient continued to develop clinically similar asymptomatic subcutaneous firm nodules largely on the right face and scalp, with a few nodules occasionally developing on the left side of his face. The nodules are self-limited, last a few weeks, and recur every few months. A timeline of the patient’s clinical presentation and documented evaluations of the recurrent deep GA from his medical record are illustrated in Figure 3.

**Figure 4:**
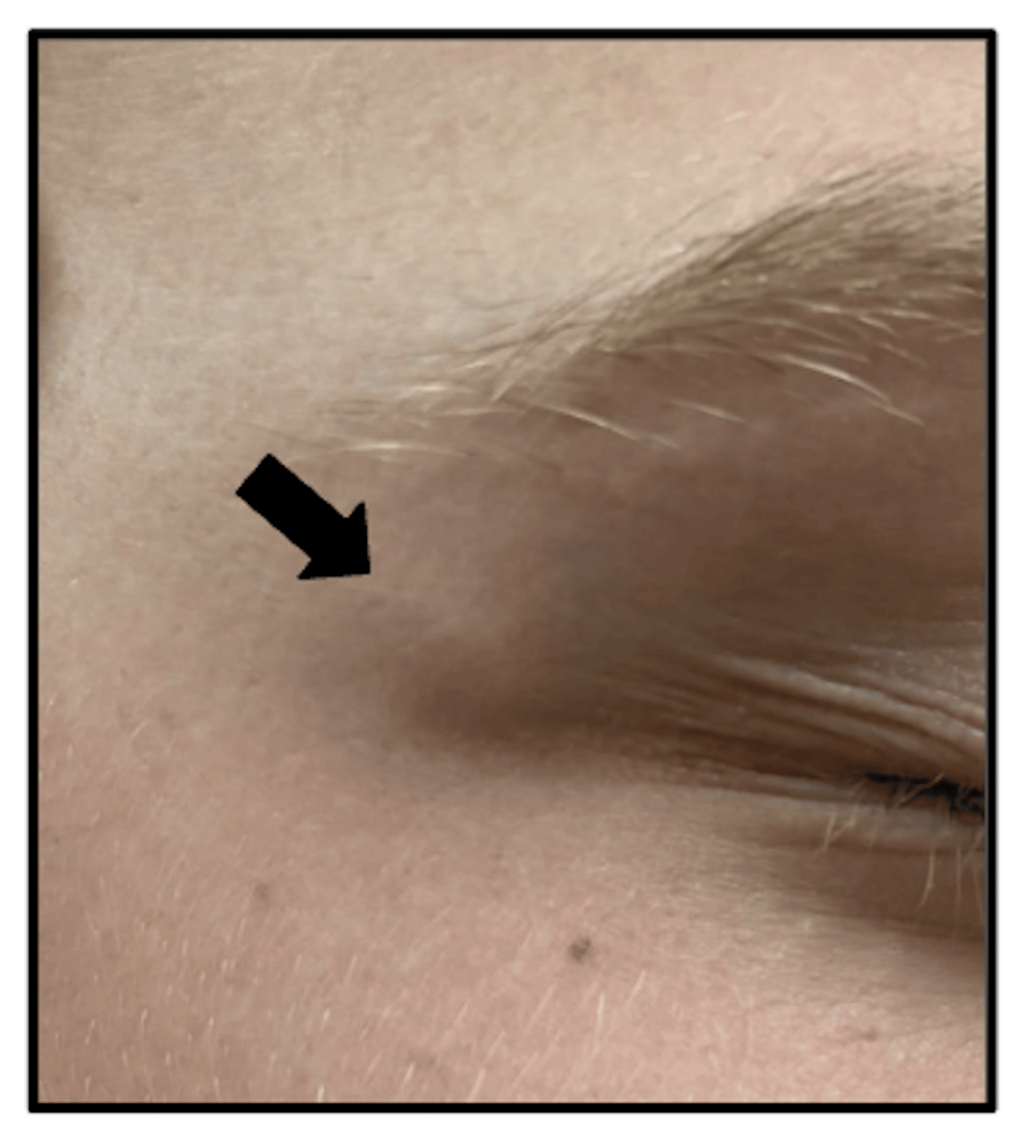
Physical exam findings The image shows a firm subcutaneous nodule on the right lateral orbital rim, consistent with deep granuloma annulare

At his last dermatology visit, the 13-year-old patient presented with several asymptomatic subcentimeter nodules scattered on the right occipital scalp, right parietal scalp, and right orbital rim (Figure 4).

Given the self-limited and asymptomatic nature of the nodules, the patient and parent elected to manage the deep GA with clinical monitoring.

## Discussion

Deep GA, also known as subcutaneous GA or pseudorheumatoid nodule, is predominantly seen in pediatric patients [2]. Among children, it is the second most common subtype after localized GA [2,5,6]. Deep GA also has the highest recurrence rate (46.5%) when compared with localized and generalized forms [5]. While the lower extremity is the most common location for all forms of GA, scalp involvement was exclusively seen in deep GA according to a systematic review of pediatric GA by Albert et al. [5]. Among pediatric patients with deep GA, the lower extremities were affected most frequently (54.9%), followed by the head and neck region (29.4%) [2]. In adult patients with deep GA, multiple nodules are more frequently observed than solitary nodules (86.8%), whereas in pediatric patients, the distribution is more balanced, with 50.6% presenting with multiple nodules [2]. 

A potential trigger for deep GA was identified in only a small proportion of pediatric cases (5.24%), with skin trauma accounting for the majority of these instances (80%) [2]. Other reported triggers include vaccinations, drugs, and postinfective reactions following respiratory tract infections [2]. This patient’s presentation is unique in that he developed multiple deep GA nodules on the head in the general distribution of a resolved scalp infection, which has not been previously reported as a trigger for deep GA in pediatric patients.

Several hypotheses have been suggested regarding the pathogenesis of Wolf’s isotopic response. One of them proposes that skin-resident memory T cells drive a delayed hypersensitivity reaction to tissue antigens altered by a preceding viral infection [7,8]. Multiple cases following herpes simplex or zoster virus infection support the role of viral triggers, with affected sites appearing more vulnerable to subsequent immune-mediated processes, including granulomatous and autoimmune conditions [7-9]. Additional support comes from a reported case of lichenoid dermatitis arising in the distribution of a prior varicella zoster virus infection, in which histopathology demonstrated increased CD45RO expression, a marker of memory T cells, in the infiltrating lymphocytes [8]. 

Lastly, the concept of an immunocompromised district, locus minoris resistentiae (LMR), has been proposed, where local damage to the immunocompetent cells, normal cell trafficking, neuromediator signaling, and lymphatic drainage can create a localized area of immunologic vulnerability [10]. LMR is usually in reference to a new development of a pre-existing condition (the Koebner phenomenon being a classic example in dermatology) [11]. While Wolf’s isotopic response is distinct from LMR with the development of a completely unrelated and distinct secondary condition, these two phenomena may share similar pathophysiology. In this patient’s case, the inflammation from the preceding localized scalp infection may have altered tissue antigens and/or immune cells in the affected area, leading to a delayed hypersensitivity reaction and/or an immunocompromised district susceptible to recurrent granulomatous dermatitis. 

The patient’s clinical history, characterized by the development of new subcutaneous nodules at the site of a previously resolved scaly dermatitis, suggests that the deep GA arose within an area of localized immune dysregulation triggered by an initial inflammatory or infectious process. To the authors’ knowledge, no prior cases of deep GA occurring at sites of previous bacterial infections in pediatric patients have been reported in the English medical literature. However, Wolf’s isotopic response has been described in association with secondary fungal and bacterial infections arising in areas of resolved dermatitis, lending further support to the concept of an immunocompromised district as the underlying pathogenic mechanism [12-14].

Our patient developed recurrent deep GA following a suspected bacterial skin infection of the scalp. This report has several limitations. The diagnosis of scalp cellulitis in this patient was presumptive, as neither cultures nor biopsies were obtained at the initial presentation, precluding definitive confirmation of both the diagnosis and the infectious source. Additionally, not every subcutaneous nodule that subsequently developed was biopsied to histologically confirm deep GA. However, both the patient and his parent consistently reported that each recurrent nodule was clinically indistinguishable from the deep GA lesion that was biopsied on the right lateral forehead. Finally, although most recurrent deep GA nodules were localized to the right side of the face and scalp, occasional lesions developed on the left side of the face. This pattern suggests the presence of an LMR that extended beyond the ipsilateral face and scalp to involve the contralateral side as well.

## Conclusions

This report highlights a rare presentation of Wolf’s isotopic response, characterized by recurrent, self-limited episodes of deep GA over a seven-year period in a pediatric patient. The temporal association between the onset of recurrent deep GA lesions and their localization to the region of prior infection and resolved scalp dermatitis suggests a possible Wolf’s isotopic response. Although this phenomenon is infrequently reported in pediatric populations - potentially due to underrecognition or underreporting - the clinical distribution in this case is consistent with previously described patterns. Most documented instances of Wolf’s isotopic response occur following herpes zoster infections, which are uncommon in children. Therefore, in pediatric patients presenting with recurrent subcutaneous nodules confined to sites of prior infection, both deep GA and Wolf’s isotopic response should be considered in the differential diagnosis.
